# Treatment Options in Gastrointestinal Cutaneous Fistulas

**DOI:** 10.1055/s-0037-1599273

**Published:** 2017-03-14

**Authors:** Itamar Ashkenazi, Fernando Turégano-Fuentes, Oded Olsha, Ricardo Alfici

**Affiliations:** 1 Department of Surgery, Hillel Yaffe Medical Center, Hadera, Israel; 2 Department of Emergency Surgery, Hospital General Universitario Gregorio Marañon, Madrid, Spain; 3 Department of Surgery, Shaare Zedek Medical Center, Jerusalem, Israel

**Keywords:** fistula, intestinal fistula, enterocutaneous fistula

## Abstract

Enterocutaneous fistulas occur most commonly following surgery. A minority of them is caused by a myriad of other etiologies including infection, malignancy, and radiation. While some fistulas may close spontaneously, most patients will eventually need surgery to resolve this pathology. Successful treatment entails adoption of various methods of treatment aimed at control of sepsis, protection of surrounding skin and soft tissue, control of fistula output, and maintenance of nutrition, with eventual spontaneous or surgical closure of the fistula. The aim of this article is to review the various treatment options in their appropriate context.

Enterocutaneous fistulas usually occur following surgery and may cause significant morbidity and mortality. They are also a source of significant misery for both the patient and caregiver. This pathology is approached in a stepwise manner, where each of the steps has both a defined goal and a defined priority. The initial steps involve control of sepsis, protection of surrounding skin and soft tissue from fistula affluent, and management of electrolyte imbalances. Nutritional support is initiated as quickly as possible. Once sepsis and fistula output are controlled, fistula anatomy is defined. This delineates which organs are involved and helps elect the most appropriate strategy of treatment.

This review describes the different treatment options emphasizing some of the less commonly used approaches in the care of this complex problem. It is emphasized that these less commonly used approaches should be regarded as complimentary tools in the arsenal of those treatments whose role is already established in the treatment of enterocutaneous fistulas. All these traditional and novel treatments and approaches involve control of sepsis, protection of surrounding skin and soft tissue, controlling fistula output, maintenance of nutrition, defining fistula anatomy, and alternative techniques for fistula closure.

## Control of Sepsis

Sepsis is the most common presenting symptom of enterocutaneous fistulas, and the majority of deaths are related to uncontrolled sepsis. 1 Computed tomography (CT) scan can define abscesses and may guide percutaneous drainage. However, some patients may need surgery, with sepsis being the most common indication for operation in this patient population. 2 During surgery, the main objective is to drain the septic focus. It is not uncommon that following such an operation, the abdominal wall is left open, leading to the formation of an enteroatmospheric fistula. 3 4

Two novel surgical options that may be applied during emergency operations are worth mentioning here. If a fistula opening is encountered and bowel cannot be repaired primarily, the “floating stoma” described by Subramaniam et al allows isolating the fistula from the rest of wound by creating a controlled stoma by suturing the edges of the hole in the intestine to the plastic silo used for temporary coverage. 5 The stoma appliance adheres well to the plastic silo. A similar solution can be applied if temporary exteriorization or proximal diversion is deemed appropriate. 6 The bowel to be exteriorized is brought out through a hole fashioned in the plastic silo and opened to drain into a stoma appliance that is adhered to the plastic silo. There are several advantages to this technique. First, it effectively facilitates proximal diversion or exteriorization in cases where abdominal sepsis has led to secondary bowel edema, mesenteric thickening, and mesenteric shortening. Another advantage of this technique is that it enables construction of a definitive stoma to be deferred to a later date when circumstances are more advantageous. In selected patients, it may allow delayed primary repair of the bowel, avoiding the need for a diverting stoma altogether. In this case, unnecessary damage to the abdominal wall and further bowel resection during reconstruction are avoided. Use of this technique should be weighed against the need to leave the abdomen open.

## Protection of Surrounding Skin and Soft Tissue

Protection of skin and surrounding soft tissue from contact with the fistula effluent is a key component to successful conservative management of enterocutaneous fistula. Enteric fluid rapidly leads to skin excoriation and breakdown. In both these fistulas and in fistulas opening into an open abdominal wound (enteroatmospheric fistula), the aim of treatment includes successful diversion of effluent away from the rest of the wound to protect the skin or surrounding soft tissue and to allow wound healing.

Skin protection has centered on the use of stoma appliances. However, these may fail if the appliance cannot adhere to the skin around the fistula opening. If the fistula opens into an open wound, stoma appliances will not adhere at all and will not be able to provide a protective environment. Placing draining catheters into the fistulas opening has been suggested. 7 8 However, this has been criticized as being counterproductive because it may enlarge the fistula opening. 3 9 One option to consider is a skin graft to the tissue around the fistula if fistula output is low or if the output can be diverted away from the open wound. 10 11

Application of negative-pressure therapy (NPT) systems to the wound and fistula opening using a dedicated vacuum-assisted closure systems has been described; however, this is controversial and is still not considered standard of care. 12 13 Initial reports describe placement of NPT on top of the wound and fistula as one unit. NPT allows the surrounding skin condition to improve while not impairing the ongoing decrease in fistula output. However, some authors discourage the use of NPT in this scenario since erosion of other bowel loops may occur, leading to the formation of additional fistulas. 14 15 16 Lowering negative pressures to as low as –25 mm Hg has been suggested to avoid this adverse event. 17

NPT may assist in healing the wound around the fistula while isolating the fistula output from the rest of the wound. It may be used to help divert the fistula output from the rest of the wound, allowing the wound around the fistula to heal and be covered with skin, which is essential for complete control of fistula output by simple stoma appliances.

Several reports describe different ways to apply NPT to the wound while isolating the fistula's output. Goverman et al cover the wound bed with petroleum jelly impregnated gauze while leaving the fistula opening uncovered. 18 Half-width specialized reticulated NPT foam is tailored to fit precisely on the wound. Again, the fistula opening is left uncovered. The polyurethane drape is placed over the NPT foam and a 2-cm hole is fashioned right above the fistula's opening. An ostomy appliance is placed over the drape where the hole was fashioned. The ostomy bag is attached to a Foley bag to enhance drainage. Continuous negative drainage is applied. Verhaalen et al modified this technique. 19 They added a barrier around the fistula opening to ensure that no effluent enters under the NPT foam. This is done using a circular piece of NPT foam around the fistula opening covered with a drape with a hole cut around the circular NPT foam. Both stoma paste and an Eakin ring (EAKIN Cohesive Seals, Convatec) are added to ensure that the rest of the wound is isolated from the fistula output. Another option described for isolating the fistula opening is to place a soft base of standard baby bottle nipple made of latex or silicone over the fistula opening. 9 The NPT foam is applied around the nipple. Again, stoma paste is added as needed to achieve a vacuum and isolate the fistula. An opening at the tip of the nipple allows the passage of a Foley catheter, which, in turn, allows gravity drainage of the fistula output.

Kearney et al described an extraperitoneal technique for closure of the fistula opening with the aid of NPT. 8 This technique was applied in four patients who suffered from low-output fistula in whom definitive operative treatment was not considered feasible. The mucosa and serosa at the fistula's opening are carefully debrided. A purse-string is placed at the fresh edge. A turnover flap of rectus abdominis fascia and a rectus abdominis muscle flap are dissected to cover the fistula opening. A Malecot catheter is introduced through the muscle and fascia into the fistula opening and secured with the purse-string suture. The catheter transforms the fistula into a controlled fistula around which the wound is covered by primary skin closure. The catheter is removed 10 to 14 days after the skin around the fistula heals.

Yet another approach is control of the fistula output itself by NPT. This can facilitate wound treatment, now devoid of persistent soiling by the fistula effluent. De Weerd et al described a method in which NPT is used to immobilize a muscle flap onto the fistula opening securing its closure. 20 NPT has also been used to control fistula output while allowing patients to return to oral nutrition. Wainstein et al treated 91 patients using pressures as high as 600 mm Hg. 21 This led to active wound contracture and compression of the fistula orifice. In 41% patients, output was suppressed within 7 days, and in 57%, output was reduced below 500 mL/day. Enteral nutrition was reintroduced within 3 to 4 days in 89 patients without significant increase in fistula output. Hyon et al describe their method in which high-pressure suction used in one patient with high-output fistula led the polymer to contract and compress against the wound bed, creating an occlusive barrier over the fistula orifice. 22 This allowed the authors to return this patient to oral diet.

NPT is associated with specific commercial devices that entail extremely high costs of treatment. Low-cost alternatives allowing application of negative pressures to wounds have been described. 23 It is questionable whether more patients treated with NPT will achieve spontaneous closure. Nevertheless, NPT may help improve wound care before surgery. 24 NPT, similar to other treatment modalities discussed, should not be judged only by its impact on spontaneous closure. It must also be emphasized that application of NPT in patients with enterocutaneous fistula is cumbersome and demands extensive nursing support.

## Controlling Fistula Output

Once enterocutaneous fistula is identified, patients are usually placed on a nil per os (NPO) regimen to diminish intestinal fluid production. Even so, normal secretion of swallowed saliva accounts for at least 0.5 L, gastric juice for 2 L, and pancreaticobiliary secretions for an additional 1.5 L. 25 Thus, interventions other than NPO may be needed to decrease gastrointestinal fluid content.

Decreasing saliva production can be achieved using anticholinergic drugs such as glycopyrrolate and scopolamine. These drugs may be of particular benefit in patients suffering from esophageal fistula. Glycopyrrolate hardly penetrates the blood–brain barrier and therefore has less neurologic side effects compared with other anticholinergic drugs. 26 27 Treatment may diminish salivary production by as much as 50%. It will also diminish gastric secretions. Side effects may include behavioral irritability, urinary retention, constipation, excessive dryness of the mouth, headache, drowsiness, blurred vision, facial flashing, vomiting, and inability to sweat in hot environments. Glaucoma is a contraindication to treatment. Possible complications of treatment should be weighed against the relative benefit.

Decreasing gastric output is possible using either proton pump inhibitors (PPI) or H2 receptor antagonists. Both inhibit gastric secretion of hydrochloric-rich fluid by parietal cells. Theoretically, PPIs should be more effective than H2 receptor antagonists since they inhibit acid secretion, whether promoted by histamine, acetylcholine, or gastrin. However, different trials examining premedication with either H2 antagonists or PPI before surgery reveal that decrease in gastric volume remnants is more pronounced following H2 antagonists. 28 Studies in other scenarios are lacking, especially those studying long-term reduction of gastric secretions. 29 Thus, whether PPI or H2 blockers are preferable in the long term is unknown.

Loperamide and codeine phosphate are antidiarrheal drugs that slow gastrointestinal transit, thus allowing more time for absorption in the proximal bowel. 25 30 The role of these two drugs is more pronounced in patients in whom oral intake is attempted. They should be given half an hour before meals. 25 Loperamide is preferred to codeine sulfate as it neither sedative nor addictive. Loperamide is reabsorbed in the terminal ileum. Thus, higher doses may be needed in patients with a small bowel fistula in whom the enterohepatic circulation is disrupted.

Administration of somatostatin and its synthetic analogues octreotide and lanreotide has been studied in patients with enterocutaneous fistula. These drugs inhibit gastrointestinal secretions and may thus have an ameliorating effect on fistula output and the resulting dehydration, electrolyte imbalance, and skin excoriation. Decreasing fistula output could aid in promoting fistula closure; however, this is controversial. 30 Though some studies documented significant reduction in fistula output, most studies to date have not shown higher fistula closure rate or shortened fistula closure time. 31 32 33 34 35 36 37 38 39 40 Data in these and other studies are confounded by the small numbers of patients included, inclusion of a large proportion of patients with pancreatic fistulas together with patients with enterocutaneous fistula, and heterogeneity within the patient cohorts studied. Data concerning the relative efficacy of one drug over another are also limited. Somatostatin must be given continuously as an intravenous injection due its short half-life. One small study has shown that both somatostatin and octreotide achieved significantly better closure rates compared with control, with the former drug achieving slightly better results than the latter drug. 38 Prolonged-release lanreotide is a synthetic analogue of somatostatin. It is administered intramuscularly, and its pharmacological effect extends as long as 10 days. One small study has shown it to reduce fistula output and to hasten closure. 41

The authors have limited experience with high-dose octreotide in patients with high-output fistula. In patients with high-output fistula, other than an NPO regimen, we add both subcutaneous octreotide and intravenous H2 blockers to inhibit gastrointestinal secretions. If no reduction in fistula output is documented, we stop the subcutaneous injections and administer octreotide intravenously in a continuous manner. This is done using a protocol developed for patients suffering from intractable diarrhea following chemotherapy. 42 Octreotide is administered as a continuous infusion of 50 μg/hour. If fistula output is not reduced considerably, dosage is increased the next day to 100 μg/hour and then to 150 μg/hour the day after. The optimal dosage is maintained for 3 to 4 days after which the dosage of octreotide is tapered off. If the output increases, the dosage is increased again. In this series, no serious adverse events were noted. 42 Commonly reported adverse events following octreotide treatment include abdominal discomfort, and pain at the injection site in case of subcutaneous injections. 43 To date, we used this protocol in only two patients, and both patients' fistula eventually closed spontaneously without surgery.

Surgery aimed at controlling fistula output by proximal diversion in cases of uncontrolled high-output fistulas has been described. 44 45 Diversion may be achieved by performing proximal ileostomy and even total disconnection of the proximal digestive tract achieved by duodenal disconnection, duodenogastrostomy, and diverting gastrostomy. These techniques should be employed selectively. Disconnection of the proximal digestive tract done through the lesser sac may be the only viable alternative in patients with inaccessible “frozen abdomen” from within which a high-output small bowel fistula has formed. Surgical diversion is achieved at the price of operative trauma to the intra-abdominal domain and abdominal wall. Proximal diversion will also increase fluid and electrolyte losses.

## Maintenance of Nutrition

Once enterocutaneous fistula is diagnosed, patients are placed on NPO as part of their initial management. Many of these patients are already malnourished due to their underlying condition and the fact that they have undergone major surgery. Nutritional supplementation should be started as early as possible since malnourishment is both a significant contributor to both mortality and failure of conservative treatment. 46 Nutritional support can be achieved enterally, parenterally, or through a combination of both. We prefer to start the patient on parenteral nutrition only. In the initial phases of treatment, our aim is to reduce bowel content as much as possible as an adjunct in treating sepsis and treating the wound, with the ultimate goal being spontaneous closure of the fistula. Whether this approach is appropriate is controversial. 47 There is a bias toward using total parenteral nutrition in those patients in whom fistula closure is expected. Thus, retrospective data purporting to show an association between total parenteral nutrition and fistula closure should be interpreted with caution. 48

Prolonged total avoidance of enteral nutrition is discouraged. Enteral nutrition promotes bowel trophism and may prevent bacteremia in selected patients. 49 50 Complications of parenteral nutrition such as line sepsis are avoided in a patient fully nourished by enteral nutrition. Thus, if the fistula fails to heal within the first weeks of presentation, and sepsis is controlled, oral intake is gradually resumed, as advocated by Hollington et al. 47

Implementation of enteral nutrition follows a trial-and-error approach. If the fistula output is low (<200 mL/day), enteral nutrition is usually well-tolerated. The patient is only allowed clear liquids at first. Fistula output is monitored as the volume of enteral nutrition is increased. The patient's electrolytes and urine output are also monitored, and intravenous electrolyte-rich fluids are supplemented as required. If the fistula output does increase, the beneficial effect of continued enteral nutrition should be weighed against the goal of reducing the fistula output as much as possible.

Spontaneous healing of a fistula commonly occurs within 6 weeks. 1 If the enterocutaneous fistula fails to heal within this timeframe, the patient will probably need surgery to close it. We aim at returning the patient to enteral intake to avoid the need for the parenteral route perioperatively. The finding of intestinal mucosa embedded within the skin or within granulation tissue of an open wound indicates that spontaneous closure of the enterocutaneous fistula is unlikely to occur. 51 In these cases, early implementation of enteral feeding should be considered.

Enteral nutrition may be administered through the opening into the efferent loop of bowel (i.e., fistuloclysis). 51 This can only be done if the tract between the skin and bowel has matured. More than 75 cm of distal small bowel is usually required for proper absorption. Proper care should be taken to fix the catheter correctly so that it is not pulled into the bowel by peristalsis. 52 Since fistuloclysis entails inserting a cannula through the bowel opening, it is therefore not the preferred option as long as spontaneous closure is deemed possible.

## Defining Fistula Anatomy

CT scan will identify an abscess and may determine which bowel segment may be implicated. If percutaneous drainage is performed, repeat CT may help identify if the abscess has been completely drained or not. CT “abcessogram” is commonly done by carefully injecting contrast material into the drain. No other contrast material is given to the patient, neither orally nor intravenously. The injected contrast will delineate the abscess cavity and may at times help delineate the fistula itself. 53 54

An alternative to contrast injection is to inject air without contrast through the draining catheter. 55 Again, no other contrast is given. Scanning is done before and after air injection. In our experience, air, compared with contrast, is more sensitive in delineating the real extent of the abscess cavity.

Once the abscess cavity is reduced in size as much as possible, fistulography is performed. The aims of this study are to define the fistulous tract, to identify the offending bowel, and to rule out distal obstruction. Traditionally, this is done using fluoroscopy. 56 CT is a more contemporary alternative and is our personal preference for fistulography.

In most patients, CT and fistulography constitute the only means needed for defining the fistula's anatomy. Special consideration should be given to patients with previous large bowel malignancy. These patients should undergo colonoscopy to rule out an occult recurrence. 57

## Alternative Techniques for Fistula Closure

Various techniques that may serve as alternative methods to surgery have been described in case series and case reports. 58 All treatments have in common the occlusion of fistula outflow while leaving the fistula intact. These are achieved either endoscopically or percutaneously. Most cases described in the literature were low-output fistula. In cases treated, fistula output usually diminished gradually and eventually closed.

Endoscopic clips or absorbable loop snares (Endoloop, Ethicon Inc., Somerville, NJ) are appropriate for colonic fistula. The opening within the colon is identified and clips are placed approximating the mucosa. Most of the experience gained has been in the acute setting of perforation. However, a few cases of established colocutaneous fistula have also been described. 59 60 61 Another endoscopic alternative is to cannulate the internal opening and to inject either N-butyl-2-cyanoacrylate (Histoacryl glue, B. Braun Medical Inc., Melsungen, Germany) or fibrin glue. 62 63 64 65 66

Glues may also be applied by percutaneous cannulation and direct injection into the fistula. There is extensive experience with fibrin glue in treatment of perianal fistulas. 67 Though closure rates are low in this setting, the relative simplicity and low morbidity make this a valuable option for treatment. This has led several authors to explore this option of treatment in patients with enterocutaneous fistula. 68 Fibrin glue is injected through the external opening until the fistula is filled. This can be done under endoscopic surveillance to ensure that the internal opening is also filled with glue. Fibrin glue can be reinjected into the tract if necessary. Some authors advocate adding a polyglactin plug (Vicryl, Ethicon Inc.) to enhance closure of fistulas originating in upper gastrointestinal tract. 69 Successful closure of fistula following percutaneous application of histoacryl has also been described in two patients with gastrocutaneous and duodenocutaneous fistula. 70 Lisle et al and Khairy et al described gelatin sponge (Gelfoam, Pharmacia and Upjohn Company, Kalamazoo, MI) embolization of the internal opening using a percutaneous approach for fistula originating from both the small bowel and the large bowel. 71 72 A guidewire is introduced through the fistula tract. An introducer sheath is placed over the guidewire, taking care to place its tip at the enteric opening of the fistula. The guidewire and enteral introducer are removed and Gelfoam pieces are pushed down the sheath to form a plug at the entrance of the fistula. Since Gelfoam is not radiopaque, it can be soaked in contrast material to aid in its deployment. 71 72 Once the plug is in place, the sheath is gently removed, taking care not to dislodge the plug. Finally, percutaneous insertion of an anal plug (Surgicis AFP, Cook Surgical) into a colocutaneous fistula has also been described. 73

For patients suffering from enteroatmospheric fistulas, several alternatives for surgery have been described. Girard et al reported a single case of one such fistula treated by patching a piece of acellular dermal matrix onto the fistula opening with fibrin glue. 74 Sarfeh and Jakowatz, and Jamshidi and Schecter described their experience with a limited number of patients in whom the edges of the fistula were sutured closed after which the suture line was buttressed with either autogenous split thickness skin graft or acellular matrix using fibrin glue. 75 76 Though success rates are limited, the risk is low and the procedure may be repeated.

## Conclusion

Approaches to the treatment of patients with enterocutaneous fistula should be individualized since this is a heterogeneous population of patients characterized by different underlying pathologies. Nonoperative treatments mentioned previously should be applied using a case-by-case approach, taking into consideration that there is no single best solution for most if not all types of fistulas. Failure with one approach does not preclude success with another. Rather, each of the aforementioned treatments is one more aid in the arsenal of treatments for this troublesome problem.
